# Supplementary material for: Functional classes of SNPs related to psychiatric disorders and behavioral traits contrast with those related to neurological disorders
Source: PLoS One. 2024 May 16;19(5):e0247212. doi: 10.1371/journal.pone.0247212 (PMC11098489; doi:10.1371/journal.pone.0247212)
Supplement: Attachment — Submitted filename: PONE-D-21-03557_reviewer.docx [file pone.0247212.s002.docx]

The paper by Reimers et al. investigated the partitioned SNP-heritability of 20 brain phenotypes by the functional classes of genomic regions including several brain-specific genomic annotations like CTCF binding sites, brain-specific promoters, splicing sites. The authors compared the relative contributions of different functional classes of genomic regions on different brain disorders/traits and compared the enrichment performance between psychiatric disorders with neurological disorders. This study presented evidence to support that most GWAS SNP is functional in regulatory with less than 13% of the genome contribute to more than 80% of the SNP heritability and highlighted the CTCF binding sites, cell type-specific enhancers, and non-coding RNA in the psychiatric disorders.

Several major concerns need to be addressed:

1. Comparing psychiatric disorders and neurological diseases is one of the major works in this study, and the authors did a good job of comparing them from the perspective of different functional classes. However, there is no justification for the selection of brain phenotypes. In a Science paper by the brainstorm consortium (PMID: [29930110](https://www.ncbi.nlm.nih.gov/pubmed/29930110)), they already compare the genetic correlation between psychiatric disorders and neurological disorders. It will be better to include all the traits in the paper to have a comparison or give justification in the selection of the phenotypes.
2. When comparing the different phenotypes, another important issue is pleiotropy, to what extend the similarity observed in this study is came from the pleiotropy is not discussed.
3. The authors highlighted that “the results provide a bridge between genetics and the well-known effects of life history and recent stressful experiences on the risk of psychiatric illness” with no direct evidence or related evaluation. It is better to change the description.

Minor concerns:

1. The figures are in low resolution.
2. The format of the tables is inappropriate.
3. The citation for LDSR is missing. The authors need to check the citation for methods.
4. The results for brain-specific CTCF are really interesting, more discussion for interpreting these results will be better.
