# Supplementary material for: Functional classes of SNPs related to psychiatric disorders and behavioral traits contrast with those related to neurological disorders
Source: PLoS One. 2024 May 16;19(5):e0247212. doi: 10.1371/journal.pone.0247212 (PMC11098489; doi:10.1371/journal.pone.0247212)
Supplement: Attachment — Submitted filename: Response to Reviews.docx [file pone.0247212.s003.docx]

August 25, 2021

Dear Editor,

As requested by the reviewers, we have included higher quality graphics files in the Figures file. To address their requests for better quality table 2, we have provided table 2 as a csv file, rather than a table in Word. We have stated more explicitly how our implication of CTCF sites links previous work on genetic contributions to psychiatric illness with work on environmental factors.

As reviewer #1 requested, we tried to include all of the traits in the BrainStorm consortium. We found that publicly reported results from several of the phenotypes were poorly suited for the LDSC tool, either because few SNPs were represented in the public databases (e.g. multiple sclerosis) or because of small case numbers (e.g. cerebral hemorrhage). Therefore we included in our analysis two additional traits  - chronic pain and stroke -  that were of sufficient quality to include in an LDSC calculation. Nevertheless we note that these additional traits are not diseases of neurons in the brain, even though they affect the brain. Therefore we reorganized our presentation of figure 2 into two new figures: a figure focused only on phenotypes likely to be due to functions of the nervous system, and one that includes our initial control traits and also the phenotypes that affect the central nervous system but do not seem to originate there.

Reviewer #2 pointed out that “the publicly available GWAS datasets sometimes cannot replicate the heritability estimates from the original study because some samples were excluded from the released version” and also suggested we combine the two GWAS for Alzheimer's. The reviewer raises a legitimate concern that the estimates, especially from a complex statistical method like LDSR, may depend sensitively on the data set or even the specific subset of data analyzed.

In this regard, the estimates from the two independent AD GWAS are expected to be even further apart than estimates based on subsets of the same data; thus these two serve as a sanity check to suggest that the profiles are robust. Therefore we would prefer to report both separately, and have explained our rationale explicitly, although the reviewer is right to point out that they count as only one trait.

Reviewer # 2 also “suggested the authors provided the same analysis in more non-psychiatric or non-neurological disorders as negative control.” We have included some other brain-related but non-psychiatric or non-neurological disorders. Our interest is primarily in brain-related disorders, and we don’t expect our brain-specific annotations to be as useful in distinguishing the functional architecture of other phenotypes.

This reviewer also requests that we comment about the possible impact of a sharing of risk variants across the psychiatric and neurologic disorders. We add a few sentences on this in the revised discussion section. We also expand on the potential reason for the placement of bipolar disorder midway between the psychiatric and neurological disorders.

Regards,

Mark Reimers and Kenneth Kendler
