# Supplementary material for: Functional classes of SNPs related to psychiatric disorders and behavioral traits contrast with those related to neurological disorders
Source: PLoS One. 2024 May 16;19(5):e0247212. doi: 10.1371/journal.pone.0247212 (PMC11098489; doi:10.1371/journal.pone.0247212)
Supplement: Attachment — Submitted filename: Response to Reviews.1-16.docx [file pone.0247212.s004.docx]

January 16, 2022

Dear Dr. Liu

In late September, during the second review of this paper, while engaged in follow-on work to our results in this paper, I found that I had inadvertently switched the labels on two columns in Figure 1: CTCF and ‘mammal conserved’. Correcting the label switch does not change the conclusions about relationships among phenotypes shown in Figure 2, but does change the conclusions shown in Figure 3, although only modestly, because the two categories that were switched both regulate inducible expression, which was our primary interpretation of the original enrichment (of CTCF). I notified the editorial assistants at PLoS One of the mistake and they replied at that I should not interrupt the review process unless the revisions would change the “scientific understanding”. They advised me to make adjustments to the submission files following editorial assessment.

Since we thought the scientific implications of the change were modest, we did not choose to interrupt the review process, but in this version I have corrected the labels in Figures 1 and 3 and changed several sentences in the results and discussion sections; I have dropped the paragraph discussing the function of CTCF. This change means that we did not respond to requests by reviewer #3 under section 6 of Review Comments to the Author.

I further tried to clarify the discussion of the relation of our results to plasticity and stress but I deleted the sentence in the abstract under Additional Editor Comments.

Regards,

Mark Reimers and Kenneth Kendler
