# Supplementary material for: Functional classes of SNPs related to psychiatric disorders and behavioral traits contrast with those related to neurological disorders
Source: PLoS One. 2024 May 16;19(5):e0247212. doi: 10.1371/journal.pone.0247212 (PMC11098489; doi:10.1371/journal.pone.0247212)
Supplement: Attachment — Submitted filename: Response to Reviews-7-18.docx [file pone.0247212.s005.docx]

Dear Editor,

My apologies again for the delays with this MS, mostly due to illness. Some amendments made in response to your requests

1. Several figures are not readable. Table 2 is chopped off. Please provide figures with good resolution.

*I remade figures from code with higher resolution.*

*I reformatted Table 2*

2. Please clarify the differences between Gen promoter and Brain promoter in figure 3. I assume that "Gen promoter" is for all gene promoter, while Brain promoter refers to brain-specific promoter. A question here is about their possible overlap (?). Is Gen promoter mostly non-specific to brain? Please provide a bit more information. A little discussion about their distinct contributions to psychiatric and neurological diseases might be useful.

*I defined 'Gen promoter' as 'generic promoter' in the Figure 1 legend, and noted no overlap with brain promoter by construction*

3. According to Fig 3, the CTCF weight is very small. How do we know that is a significant contributor? Moreover, since this CTCF is from PsychENCODE, it should be labeled as "brain CTCF," which could be very different from other tissue CTCF.

*The discriminant analysis does not readily return a measure of significance for each predictor, although this could be estimated indirectly by leave-one-out analysis. For dimensionless measures, as is the case here, the size of discriminant coefficient for a predictor is normally taken as a measure of its importance. Therefore, I included all bars in the new Figure 3, so readers may easily judge the relative importance.*

Regards,

Mark Reimers
