# Supplementary material for: Functional classes of SNPs related to psychiatric disorders and behavioral traits contrast with those related to neurological disorders
Source: PLoS One. 2024 May 16;19(5):e0247212. doi: 10.1371/journal.pone.0247212 (PMC11098489; doi:10.1371/journal.pone.0247212)
Supplement: Attachment — Submitted filename: Response to Reviews-2022-9-11.docx [file pone.0247212.s006.docx]

Dear Editor

In response to your requests (numbered and copied below) about the last submission, we have done the following:

*1. Please discuss the distinction of Gene promoter and brain promoter in Fig 3? Psychiatric disorders and neurological diseases are both brain-related. How could they be different? Is gene promoter all non-brain? Is it suggesting that neurological diseases have non-brain gene promoters affected? Sounds odd.* 

We have distinguished more clearly generic promoter and brain promoter in the text

Since several of the neurological disorders heavily involve the immune system, (both microglia and cardiovascular events for stroke), think it is understandable that the generic promoters contribute more to those disorders. Even those neurologic disorders that are largely brain based on their pathology – Parkinson’s Disease, Alzheimer’s disease and epilepsy –seem to involve cellular pathologies quite different than the psychiatric disorders, which seem to involve more subtle imbalances. We have added a sentence to this effect in the Discussion.

*2. The PsychENCODE brain CTCF is used in the analysis. Should we have a non-brain CTCF used for comparison too? The author had ENCODE CTCF before but dropped it in the revision. Why?
I am also surprised to see that brain CTCF was enriched in psychiatry in the previous version but was enriched in neurological diseases in the revised version (brain CTCF has a negative value, as shown in Fig 3). What’s changed to make such a switch?*

In the first two versions of this paper that we submitted, we had mistakenly switched the labels of columns for brain CTCF, which was corrected in the most recent two versions. We highlighted this change in the response letter for the third submission. That accounts for the switch in CTCF. The previous label was ‘CTCF peaks’, which has been re-labeled as brain CTCF in order to address a previous reviewer’s comments. These CTCF peaks came from PsychENCODE (different from ENCODE) as noted in Table 1.

*3. There are several jumps from “brain promoter + conserved regions” to “enhancer” to “stress response in minority cell types.” Where is the evidence? Data? It seems to be more speculation.*

We argued explicitly in the text that a large fraction of conserved regions which are not explicitly identified as coding or promotor regions would be enhancers. It is thought that most enhancers are attachment points for specific DNA binding proteins, which are typically induced by particular cell signals. While there are many developmental enhancers, in the adult it is thought that most signals reflect a departure from homeostasis, hence a ‘stress’ in the generic sense. We think that various organismic stressors, such as infection, poverty, neglect or violence, will constitute departures from homeostasis. We have flagged this last conclusion as somewhat speculative in the Discussion.

Regards,

Mark Reimers and Kenneth Kendler
