# Supplementary material for: Functional classes of SNPs related to psychiatric disorders and behavioral traits contrast with those related to neurological disorders
Source: PLoS One. 2024 May 16;19(5):e0247212. doi: 10.1371/journal.pone.0247212 (PMC11098489; doi:10.1371/journal.pone.0247212)
Supplement: Attachment — Submitted filename: Response to Reviews 2022-11-25.docx [file pone.0247212.s007.docx]

Dear Editor

*In the introduction, the authors stated “We further expected long non-coding RNAs (lncRNAs) to contribute to psychiatric disorders because they were highly expressed in specific brain cell types and play critical roles during development.” So, this seems to be one of the two primary goals of this paper.*

*In the discussion, the authors had “On the theory of a developmental origin for psychiatric disorders, we expected to find substantial heritability for psychiatric syndromes in lncRNAs expressed during early development. Indeed, we found that all classes of noncoding RNAs appeared enriched across all phenotypes, consistent with the emerging idea that non-coding RNAs play a role in human brain diseases”.*

*But none of the results is related to lncRNA or early development. Where is the literature to claim lncRNA is early-development-specific? I have no clue how the authors came to this conclusion.*

*In the results, the authors showed: “We found that the three classes of non-coding RNAs tested (miRNAs, lncRNAs, rRNAs) appeared greatly enriched (medians 20-fold, 12-fold, and 20-fold, resp.) for contributions to SNP-heritability.” This is not for lncRNA only. I don’t see how it can be related to early development. There is no clear logic for me to follow. Moreover, there is not result shown for non-coding RNA enrichment, other than this description. If this is one of the two major goals, how could they have not even data to show?*

Although several papers have argued for the importance of long non-coding RNA's in early brain development (e.g. PMID: 27081004; PMID: 32554106; PMID: 31698782; PMID: 30728830), this claim is not at present well-established in the field. As you point out we do not actually present any data or results about long non-coding RNA’s, or indeed, any non-coding RNA's, because of wide error bars and hence insufficient statistical power. Therefore, we have deleted the discussion of enrichment and biological roles of non-coding RNA's, and kept only the technical mention of lack of statistical power, so that interested readers will know why they are not included.
